# Characterization of a rhodanese homologue from *Haemonchus contortus* and its immune-modulatory effects on goat immune cells *in vitro*

**DOI:** 10.1186/s13071-020-04333-6

**Published:** 2020-09-07

**Authors:** Yujian Wang, Muhammad Ehsan, Jianmei Huang, Kalibixiati Aimulajiang, RuoFeng Yan, XiaoKai Song, LiXin Xu, XiangRui Li

**Affiliations:** 1 School of Life Science, Huizhou University, Huizhou, 516007 People’s Republic of China; 2 MOE Joint International Research Laboratory of Animal Health and Food Safety, College of Veterinary Medicine, Nanjing Agricultural University, Nanjing, 210095 People’s Republic of China

**Keywords:** *Haemonchus contortus*, Rhodanese, Peripheral blood mononuclear cell (PBMC), Immunomodulation

## Abstract

**Background:**

Modulation of the host immune response by nematode parasites has been widely reported. Rhodaneses (thiosulfate: cyanide sulfurtransferases) are present in a wide range of organisms, such as archaea, bacteria, fungi, plants and animals. Previously, it was reported that a rhodanese homologue could be bound by goat peripheral blood mononuclear cells (PBMCs) *in vivo*.

**Methods:**

In the present study, we cloned and produced a recombinant rhodanese protein originating from *Haemonchus contortus* (rHCRD), a parasitic nematode of small ruminants. rHCRD was co-incubated with goat PBMCs to assess its immunomodulatory effects on proliferation, apoptosis and cytokine secretion.

**Results:**

We verified that the natural HCRD protein localized predominantly to the bowel wall and body surface of the parasite. We further demonstrated that serum produced by goats artificially infected with *H. contortus* successfully recognized rHCRD, which bound to goat PBMCs. rHCRD suppressed proliferation of goat PBMCs stimulated by concanavalin A but did not induce apoptosis in goat PBMCs. The production of TNF-α and IFN-γ decreased significantly, whereas secretion of IL-10 and TGF-β1 increased, in goat PBMCs after exposure to rHCRD. rHCRD also inhibited phagocytosis by goat monocytes. Moreover, rHCRD downregulated the expression of major histocompatibility complex (MHC)-II on goat monocytes in a dose-dependent manner, but did not alter MHC-I expression.

**Conclusions:**

These results propose a possible immunomodulatory target that may help illuminate the interactions between parasites and their hosts at the molecular level and reveal innovative protein species as candidate drug and vaccine targets.
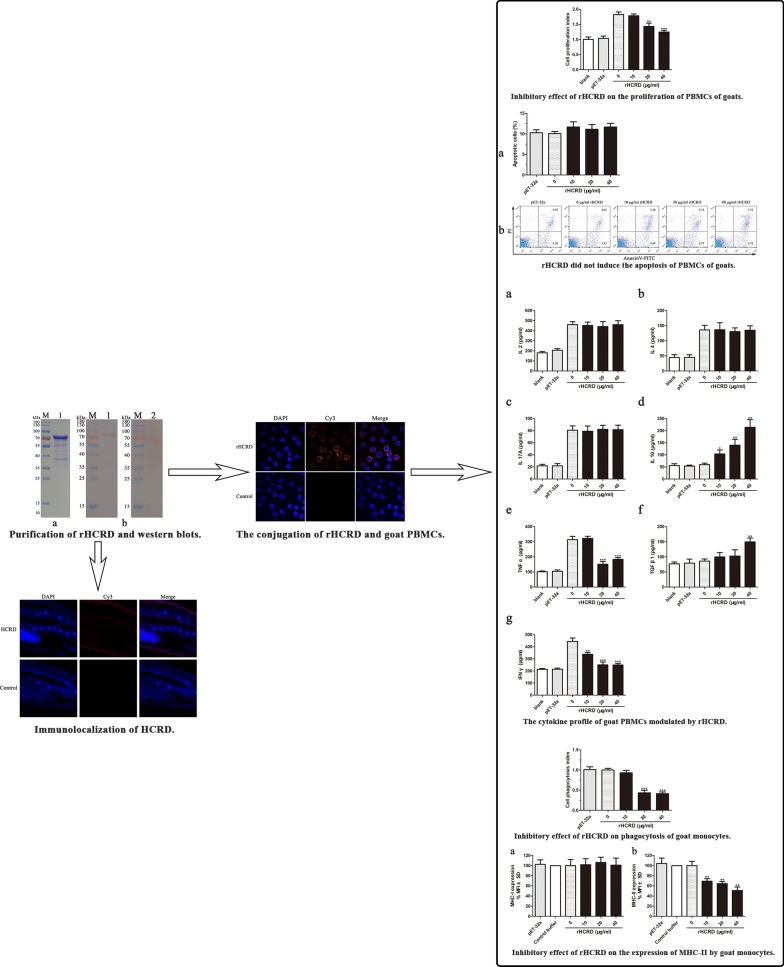

## Background

The parasitic nematode *Haemonchus contortus* typically occurs in small ruminants and is an especially severe threat to the production and health of goats and sheep in warm and tropical temperate zones [1]. Specialized and highly complex interactions between parasitic nematodes and their hosts involve the parasite escaping from immunological responses, which allows the parasite to survive within its host [2, 3]. In order to evade the host immune system, a variety of molecules produced by nematode parasites are released as excretory/secretory proteins and/or located on the cuticle surface at the host-parasite interface [4, 5].

Rhodaneses (thiosulfate: cyanide sulfurtransferases) are present in a wide range of organisms, including archaea, bacteria, fungi, plants and animals [6, 7]. Rhodanese homology domains (RHDs), which have a C-terminal domain possessing a cysteine residue at the active-site, are ubiquitous structural modules [8]. Furthermore, a superfamily of variant rhodaneses exists that is composed of single or tandem RHDs or combined with other protein domains with different functions [9]. *In vitro*, rhodanese catalyzes the irreversible transfer of a sulfane sulfur atom from a suitable donor (i.e. thiosulfate) to cyanide, leading to the formation of less toxic sulfite and thiocyanate [10]. During catalysis two separate sulfur-transfer steps are believed to occur. In the first step, the thiosulfate (S_2_O_3_^2-^) anion is attacked by the sulfhydryl (-SH) group of the conserved cysteine residue, forming a covalent persulfide intermediate. In the second step, a cyanide (CN^-^) ion attacks the persulfide intermediate, which releases the thiocyanate (SCN^-^) product and regenerates the cysteine sulfhydryl group [6, 11]. Rhodanese is thought to play a key role in cyanide detoxification [12–16]. Thus, it is necessary for herbivorous and omnivorous mammals who eat plants that contain cyanogenetic glucoside. Previously, it was reported that levels of rhodanese in different tissues and organs of mammalian animals are correlated with exposure to cyanide [12, 15, 17, 18]. In addition to cyanide detoxification, a growing body of evidence suggests that rhodaneses and RHD-containing proteins are involved in many other physiological processes, including iron-sulfur cluster assembly and regulation of oxidative phosphorylation [19–21], maintenance of the sulfane pool [7], maintenance of redox homeostasis [22], selenium metabolism [23, 24], thiamin biosynthesis [25], assisted protein refolding [26, 27], regulation of the cell cycle [9], aging-related regulation [28], molybdenum cofactor biosynthesis [29], protection of Fe-S enzymes against oxidative damage [30], xenobiotic-induced oxidative-stress and detoxification [31, 32].

Baghshani et al. [33] reported that at least some rhodanese activity was present in seven investigated parasitic helminths, including *Haemonchus longistipes*, although its activity was much lower than the values previously reported for some tissues of their vertebrate hosts. The role of rhodanese in parasitic helminths is fascinating given its wide distribution. Previously, Gadahi et al. [34] reported that excretory and secretory products of *H. contortus* could exert immunomodulatory effects on goat peripheral blood mononuclear cells (PBMCs) *in vitro* and subsequently identified a series of excretory and secretory proteins capable of binding to goat PBMCs *in vivo* [35], including an *H. contortus* rhodanese (HCRD).

In the present study, an *H. contortus* rhodanese gene was cloned and the recombinant *H. contortus* rhodanese protein (rHCRD) was expressed to analyze its immunomodulatory effects on goat PBMC and monocyte functioning using various techniques.

## Methods

### Parasites, animals and cells

The *H. contortus* strain Nanjing 2005 was acquired from Nanjing in Jiangsu province, China. Helminth-free goats aged 3–6 months-old were used to maintain the strain through continuous passage [36]. Experimental animals were challenged with stage 3 larvae (L3), which were isolated from the feces of goats given monospecific infections, cultured at 26 °C and preserved in water at 4 °C at a larval concentration of 2500 per ml.

Native mongrel male goats aged 3–6 months-old were acquired from a herd maintained for study and education at Nanjing Agricultural University and kept in indoor pens containing 6 goats per pen. The goats were provided with cured hay and whole grain maize and allowed to drink water freely. Levamisole at a dose of 8 mg per kg body weight was given peros (p.o.) every 14 days to expel naturally-occurring strongylids. After 14 days, helminth eggs were collected from goat feces under a light microscope using standard parasitology techniques. Goats confirmed to be free from nematode infections were utilized to conduct the following experiments. Goats were observed every day throughout the study to ensure their health.

Sprague-Dawley rats weighing approximately 150 g were purchased from the Experimental Animal Center of Jiangsu, Yangzhou, China (qualified certificate: SCXK 2008-0004), maintained in a sterile environment and supplied with sterile food and water.

The standard Ficoll-Hypaque (GE Healthcare, Little Chalfont, UK) gradient centrifugation method was used to separate PBMCs from whole blood with added heparin, and the obtained cells were washed twice with phosphate buffered saline (PBS) [37]. The cell density was then adjusted to 1 × 10^6^ cells/ml and cells were cultured in RPMI 1640 (Gibco, Grand Island, New York, USA) containing 100 mg/ml streptomycin (Gibco), 100 U/ml penicillin (Gibco) and 10% heat-inactivated fetal calf serum (Gibco) in a humidified cell chamber at 37 °C and 5% CO_2_. Trypan blue dye was used to determine cell viability, which was > 95% for all relevant experiments.

To obtain goat monocytes, a 6-well flat-bottom tissue culture plate (Corning, New York, USA) was used to culture goat PBMCs with RPMI 1640 culture medium (Gibco) supplemented with 10% fetal calf serum (Gibco), 100 U/ml penicillin and 100 mg/ml streptomycin (Gibco). Plates were incubated at 37 °C in a humidified atmosphere with 5% CO_2_ for 1 h. Non-adherent cells were drained by washing twice with PBS. The cells (monocytes) stuck to the bottom of the plate were collected and adjusted to a density of 1 × 10^6^ cells/ml, and viability was confirmed to be > 95% in all preparations using a trypan blue exclusion test.

### Cloning of HCRD and bioinformatics analyses

Based on the sequence of the open reading frame (ORF) of the rhodanese-like gene retrieved from the online database (GenBank: CDJ82729.1), the following primers were designed to specifically amplify the gene using reverse transcription-polymerase chain reaction (RT-PCR): forward primer (5’-ACG GAT CCA TGA TGT GTC CAC CTC CA-3’) and reverse primer (5’-GCA AGC TTG GAG AAC TGT AAC TGC CT-3’). The underlined sequences indicate *BamH*I and *Hind*III restriction endonuclease sites. The RT-PCR products were ligated with pMD19-T vector (Takara, Dalian, China) to produce pMD-rhodanese. Fragments of rhodanese were then cleaved from the pMD-rhodanese plasmid using *BamH*I and *Hind*III before being subcloned into the relevant location of the pET32a vector (Invitrogen, Carlsbad, CA, USA). Sequencing analysis was used to confirm proper plasmid construction.

### Expression and purification of rHCRD in *Escherichia coli*

To express the recombinant fusion protein, isopropyl-β-D-thiogalactopyranoside (IPTG) at a final concentration of 1 mM was used to induce *Escherichia coli* BL-21 cells (DE3) cultured in Luria-Bertini (LB) medium containing 100 μg/ml ampicillin at 37 °C for 6 h. A His•Bind^®^ 128 Resin Chromatography kit (Novagen, Madison, WI, USA) was used to purify the histidine (His)-tagged fusion protein from the precipitated bacterial lysate according to the manufacturer’s instructions. The refolded protein was purified in renaturation buffer (20 mM Tris-Cl, 500 mM NaCl, 1 mM reduced glutathione and 0.1 mM oxidized glutathione, pH 8.0) containing different concentrations of urea (0, 2, 4, 6 or 8 M) and dialyzed using PBS (pH 7.4). The empty pET32a vector was used to produce a control His-tagged protein, which was expressed and purified using an identical procedure to that used to produce the rhodanese-His-tagged fusion protein. Subsequently, 12% sodium dodecyl sulfate-polyacrylamide gel electrophoresis (SDS-PAGE) and Coomassie bright blue staining were used to assess the purity of rHCRD after purification, and the Bradford method was used to quantify protein samples. Detoxi-Gel Affinity Pak prepacked columns (Thermo Fisher Scientific, Waltham, MA, USA) were used to deplete lipopolysaccharide from the obtained rHCRD protein, and the concentration of the recombinant protein samples was adjusted to 1 mg/ml before performing limulus amebocyte lysate (LAL) assays. A Pyrosate^®^ Kit (Cape Cod Inc., East Falmouth, MA, USA) was used for LAL gel clot assays to measure endotoxin units (EU) in protein samples. Only samples with endotoxin levels lower than 1 EU/mg recombinant protein were used in further experiments.

### Generation of polyclonal antibodies

Five goats were used to produce antisera against *H. contortus*, which was used for western blots. A total of 5000 L3 larvae with infective activity was administered p.o. to goats who were maintained in a helminth-free environment. After 30 days, antiserum samples were collected and preserved at − 70 °C for further use.

In order to generate polyclonal antibodies against rHCRD, a mixture of Freund’s complete adjuvant and 0.3 mg purified rHCRD was used to inoculate Sprague-Dawley rats by subcutaneous injection at a series of sites, based on the protocol proposed by Wang et al. [38]. The rats received an initial immunization followed by four booster immunizations of the same dose at intervals of 14 days. Ten days after the final immunization, antiserum, which contained specific antibodies against rHCRD, was collected, and its reactivity was determined using an enzyme-linked immunosorbent assay (ELISA).

### Western blot analysis

Protein samples containing 20 μg purified rHCRD were isolated by 12% SDS-PAGE, and the protein bands in the gel were transferred onto Hybond-C Extra nitrocellulose membranes (Amersham Biosciences, London, UK). The membranes were immersed in blocking buffer containing 5% skimmed milk and Tris-buffered saline (TBS) for 1 h under ambient conditions to block non-specific binding sites. Subsequently, TBS containing 0.1% Tween-20 (TBST) was used to wash the membranes five times for 5 min each. The primary antibody (i.e. antiserum obtained from goats experimentally infected with *H. contortus*) was diluted 1:100 in TBST and used to incubate the membranes for 1 h at 37 °C. The membranes were then washed five times with TBST and treated with horseradish peroxidase (HRP)-conjugated rabbit anti-goat IgG (Sigma-Aldrich, St. Louis, MO, USA) diluted 1:2000 in TBST for 1 h at 37 °C. Finally, a fresh preparation of diaminobenzidine (DAB, Sigma), which acted as a chromogenic reagent, was added for 5 min to visualize the immune reaction.

### Localization of HCRD by immunohistochemistry

Adult nematodes were immersed in TISSUE-TeK^®^ O.C.T. compound (Sakura, Torrance, CA, USA), washed with PBS and immersed in PBS containing 0.2% glutaraldehyde and 4% formaldehyde for 1.5 h. The nematodes were then flash frozen in liquid nitrogen and stored at -20 °C for further use. Cryostat sections with a thickness of 10 μm were washed with PBS and immersed in PBS containing 10% normal goat serum in order to block non-specific binding sites. Subsequently, serum from rats immunized with rHCRD diluted to 1:100 was used to incubate separate sections for 1 h at 37 °C. Serum from normal rats was used as the control. The sections were then washed three times with PBS for 15 min and treated with Cy3 goat anti-rat IgG (ab6953; Abcam, Cambridge, MA, USA) for 1 h. Finally, DAPI (Beyotime, Haimen, Jiangsu, China) and PBS were used to stain and wash the sections, respectively, and anti-fade fluoromount solution (Beyotime) was applied to prevent fading as the samples were observed under a fluorescent microscope.

### Binding of rHCRD to goat PBMCs

Goat PBMCs were isolated immediately prior to experiments as described above, inoculated into 24-well plates and cultured with 40 μg/ml rHCRD on glass cover slides for 1 h at 37 °C. Untreated cells were used as controls. Then, 0.1 M PBS was used to wash the slides, and 4% paraformaldehyde was used to fix the slides under ambient conditions for 30 min. Subsequently, PBS containing 5% normal goat serum was used to incubate the slides to block non-specific binding sites, and rat polyclonal antibody against rHCRD diluted 1:100 in PBS containing 5% normal goat serum was used to incubate the slides overnight at 4 °C. Cy3 goat anti-rat IgG (ab6953, Abcam) diluted 1:400 in PBS containing 5% normal goat serum was used to incubate the slides at 37 °C for 1 h before counterstaining with DAPI (Beyotime). The nucleus and rHCRD were indicated by blue and red, respectively, under scanning confocal laser microscopy (LSM710; Zeiss, Jena, Germany) with an oil immersion lens. Images were acquired by selecting the blue and red color channels corresponding to DAPI and Cy3, respectively. The fluorescent microscope settings used for observing control samples were identical to those used for rHCRD-treated cells. Unstained controls were used to detect background staining and auto-fluorescence of the protein and cells. ZEN software (Zeiss) was used to perform synergistic combinations and for photo acquisition. Observations were independently collected from three individual samples.

### Cell proliferation assays

Concanavalin A (ConA, 10 μg/ml) was used to activate goat PBMCs at the same time as they were incubated with a range of concentrations of rHCRD at 37 °C and 5% CO_2_ for 72 h. Cell counting kit-8 assay reagent (Beyotime) was added into each well of a 96-well plate and the OD450 was measured using a microplate reader (Thermo Fisher Scientific) after incubation for 4 h. The OD450 of control cells in the blank group was set as 100%. The following equation was used to calculate the proliferation index of the cells: OD450 sample/OD450 control.

### Apoptosis assay

Flow cytometry was used to analyze cell apoptosis, as previously described [39]. Briefly, a range of concentrations of rHCRD was used to culture goat PBMCs. Control cells received no rHCRD treatment. Subsequently, the cells were stained with annexin V and propidium iodide (PI; Miltenyi Biotec, Bergisch Gladbach, Germany) according to the manufacturer’s instructions.

### Detection of cytokine secretion

To assess cytokine secretion, 10 μg/ml of ConA with or without rHCRD was used to stimulate goat PBMCs for 72 h. After collecting the supernatant, cytokines were measured using ELISAs. Commercial goat ELISA kits (Mlbio, Shanghai, China) were used to determine the concentrations of IL-2, IL-4, IL-10, IL-17A, TNF-α, IFN-γ and TGF-β1 in the supernatant of samples. Data obtained from three individual experiments were used to perform the analyses.

### FITC-dextran internalization

The phagocytic ability of goat monocytes in response to rHCRD was determined by FITC-dextran internalization followed by flow cytometry analysis (BD Biosciences). Monocytes were treated with rHCRD for two days and then incubated with FITC-dextran (1 mg/ml in RPMI1640) for 1 h at 37 °C. Cells incubated with an equal concentration of FITC-dextran were used as the baseline for monocyte phagocytosis. Finally, cells were washed twice to eliminate excess FITC-dextran and results were analyzed using FlowJo 7.6 software (Tree Star, Ashland, OR, USA) with the median fluorescence intensity (MFI) values of the control set to 100%.

### Analysis of major histocompatibility complex molecule expression

The purified monocytes (0.5 × 10^6^ cells/ml) were poured into 24-well culture plates containing complete RPMI 1640 and different concentrations of rHCRD or equal volumes of control buffer for 24 h at 37 °C. Afterward, monocytes were marked with monoclonal major histocompatibility complex (MHC)-I (MCA2189A647) and MHC-II (MCA2226F) antibodies (AbD Serotec, BioRad Laboratories, CA, USA). The results were expressed as the percentage of MFI and analyzed on a FACS Calibur cytometer (BD Biosciences).

### Statistical analysis

The data are represented as average ± standard deviation. Analysis of variance was used to calculate significant differences and Student’s t-tests were used to evaluate parametric samples (GraphPad Prism, San Diego, CA, USA).

## Results

### Cloning and sequencing of the HCRD gene

In a search for the rhodanese gene in online databases, a homologous *H. contortus* HCRD gene was identified. The HCRD protein is composed of 443 amino acid residues and has an isoionic point of 8.26 and a predicted molecular weight of 50.6 kDa. A conserved rhodanese homology domain was detected in the putative amino acid sequence (positions 220–336). No signal peptide was identified by analysis of amino acids using the SignalP program.

### Expressing and purifying HCRD

**Fig. 1:**
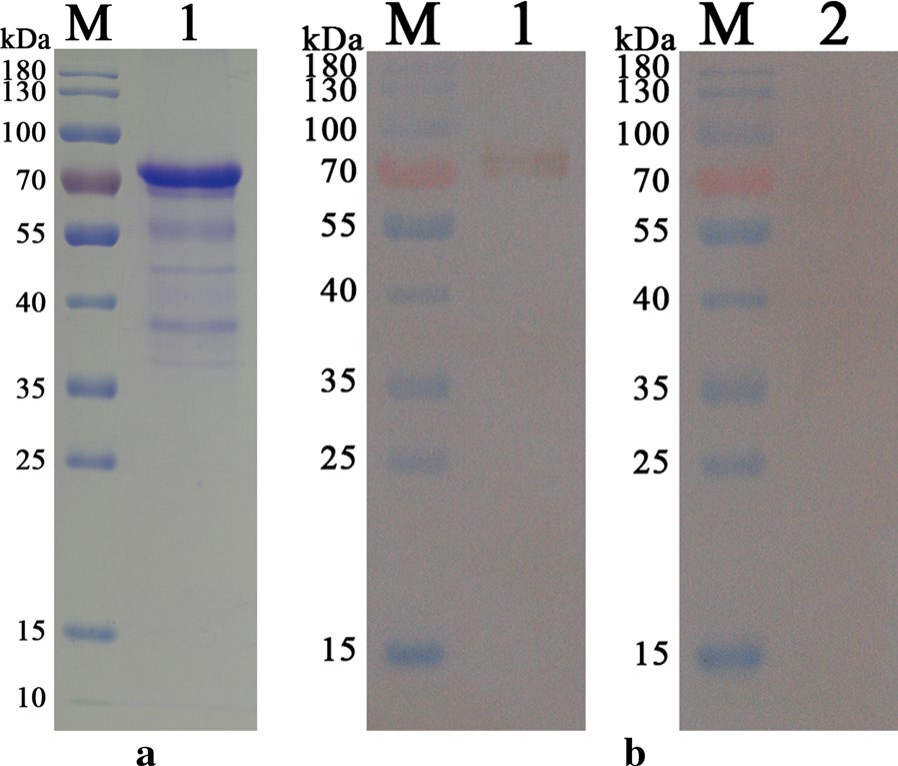
Purification of recombinant rhodanese protein from *Haemonchus contortus* (rHCRD) and western blots. **a** Separation of purified rHCRD by SDS-PAGE using a 12% polyacrylamide gel and Coomassie brilliant blue R250 staining. **b** Western blots of rHCRD after purification. Antisera obtained from goats experimentally infected with *H. contortus* was used as the primary antibody to recognize the protein (Lane 1), whereas sera from uninfected goats was used as the control (Lane 2)

By ligating the HCRD gene into the pET32a plasmid, a recombinant protein with two His 6 tags was successfully expressed in *E. coli*, resulting in a protein with a predicted molecular weight of 70.6 kDa (Fig. 1a). SDS-PAGE results showed that the purity of the rHCRD was > 90%.

### Western blots

Western blot analysis showed that rHCRD was recognized by the serum of goats subjected to experimental *H. contortus* infection (Fig. 1b), indicating that HCRD was exposed to host immunity in the process of infection.

### Immunolocalization of HCRD

**Fig. 2:**
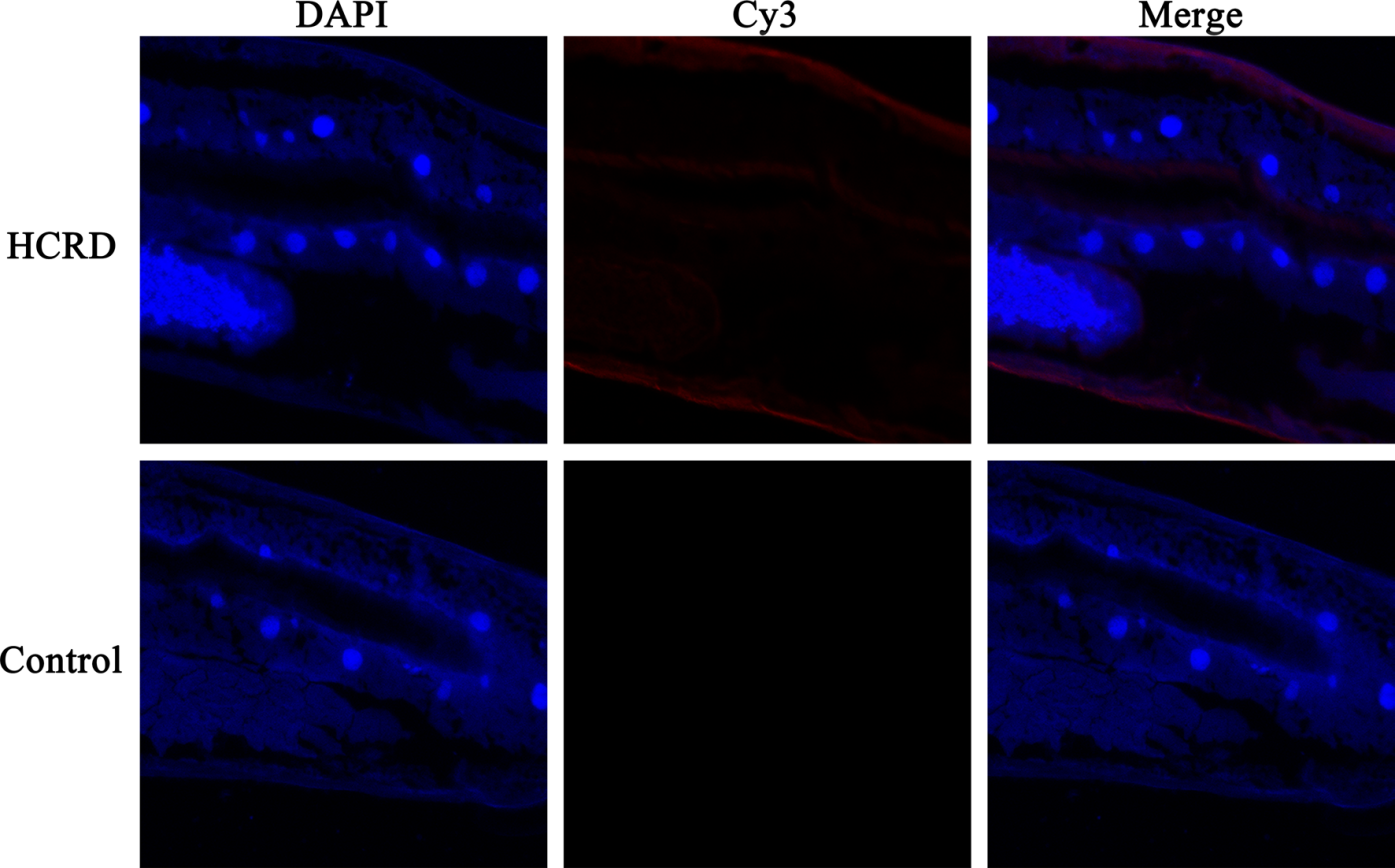
Immunohistochemical localization of native rhodanese protein from *Haemonchus contortus* (HCRD) in frozen sections of *H. contortus*. Cy3-labelled goat anti-rat IgG (ab6953, Abcam) was used as the secondary antibody to detect HCRD protein by indirect immunofluorescence. DAPI was used to counter-stain sections in order to observe DNA

Figure 2 shows a longitudinal section of a female worm in which blue and red fluorescence indicate DNA and HCRD, respectively. The predominant binding sites of antibodies eluted from rHCRD included the inner surface of the intestinal wall and the body surface, as shown in Fig. 2. Control sections exhibited no fluorescent signal.

### Binding of rHCRD to goat PBMCs

**Fig. 3:**
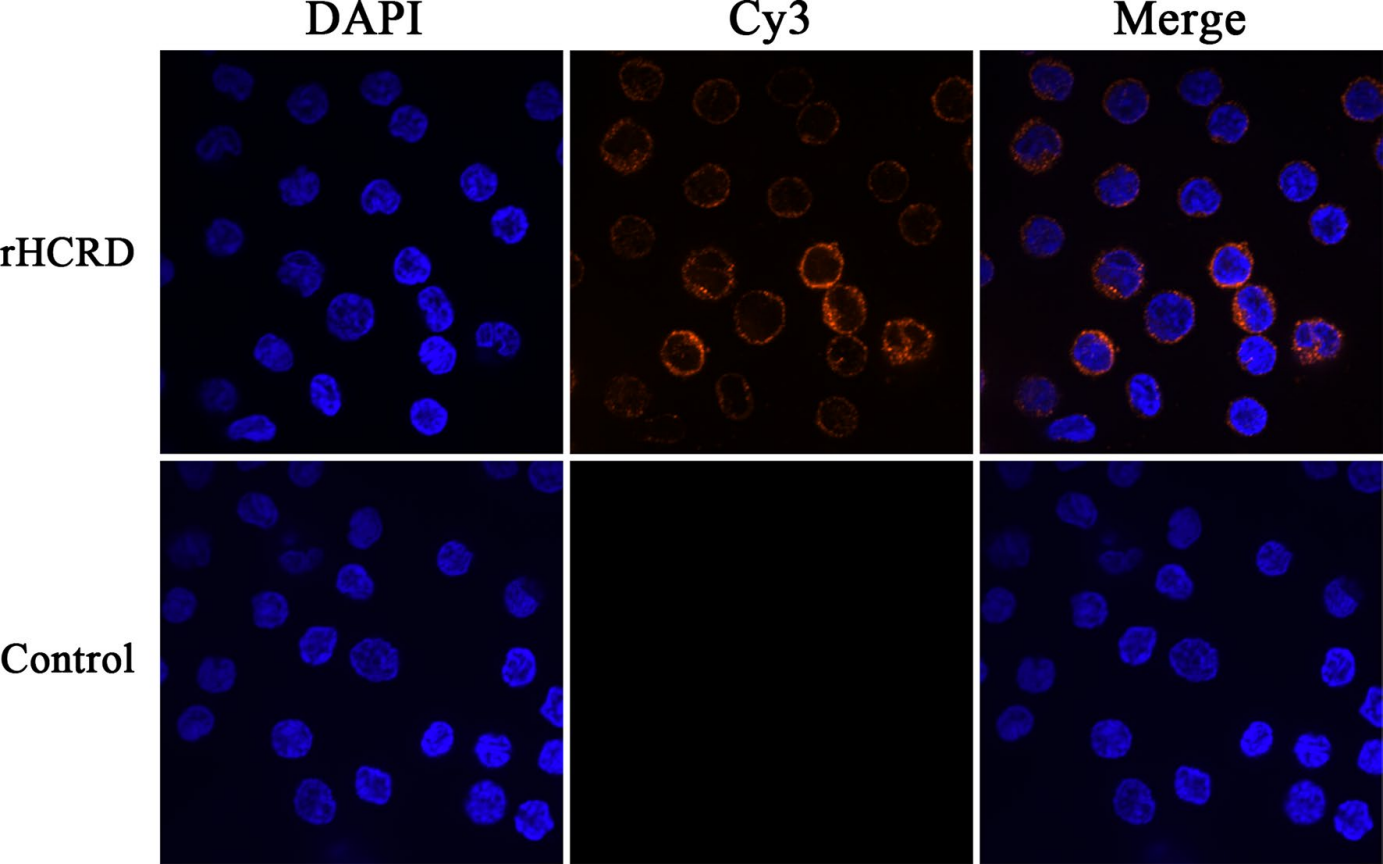
Conjugation of recombinant rhodanese protein from *Haemonchus contortus* (rHCRD) and goat peripheral blood mononuclear cells (PBMCs). Goat PBMCs were treated with rHCRD (40 μg/ml) or untreated at 37 °C for 1 h. After fixing, the cells were incubated with rat anti-rHCRD antibody followed by Cy3-labelled goat anti-rat IgG (red), and DAPI (blue) staining was used to visualize the nuclei. A confocal laser scanning microscope was used to visualize the binding of rHCRD and goat PBMCs. The overlapping blue and red channels were merged. Tests were independently performed in triplicate

As shown in Fig. 3, blue and red fluorescence indicate the nucleus stained with DAPI and rHCRD labelled with Cy3, respectively. The unstained background control exhibited no fluorescence in any color channel (data not shown). As shown in the lower panel of Fig. 3, no red fluorescence signal was observed in controls, whereas a strong red fluorescence signal was observed in cells that were treated with rHCRD, as shown in the upper panel of Fig. 3. These results indicate that rHCRD could be bound by goat PBMCs.

### Cell proliferation assay

**Fig. 4:**
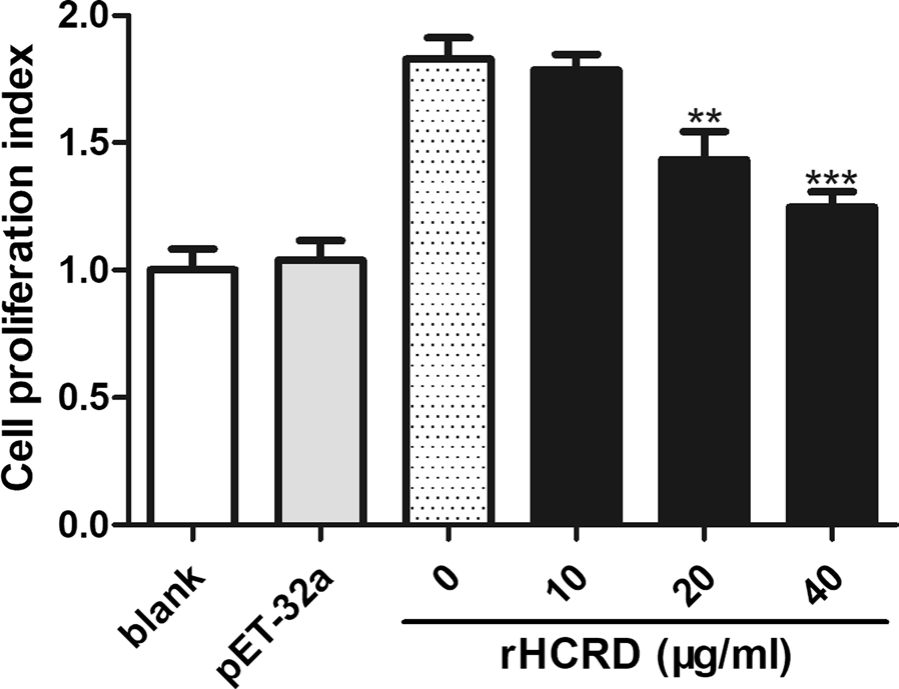
Inhibitory effect of recombinant rhodanese protein from *Haemonchus contortus* (rHCRD) on goat peripheral blood mononuclear cell (PBMC) proliferation. ConA (10 μg/ml) was used to stimulate goat PBMCs for 72 h with or without a range of concentrations of rHCRD and His-tagged protein. CCK-8 incorporation was used to measure proliferation and the cell proliferation index was calculated based on the assumption that the absorbance at 450 nm of the blank group was 100%. Tests were independently performed in triplicate. (*P < 0.05, **P < 0.01, ***P < 0.001)

Cell proliferation analysis revealed that rHCRD remarkably inhibited ConA-induced proliferation of goat PBMCs in a dose-dependent manner (10 μg/ml: *t*_(4)_ = 0.7383, *P* = 0.5013; 20 μg/ml: *t*_(4)_ = 5.006, *P* = 0.0075; 40 μg/ml: *t*_(4)_ = 9.939, *P* = 0.0006), but there was no significant change in the His-tag protein-treated group (Fig. 4).

### rHCRD did not induce apoptosis of PBMCs of goats

**Fig. 5:**
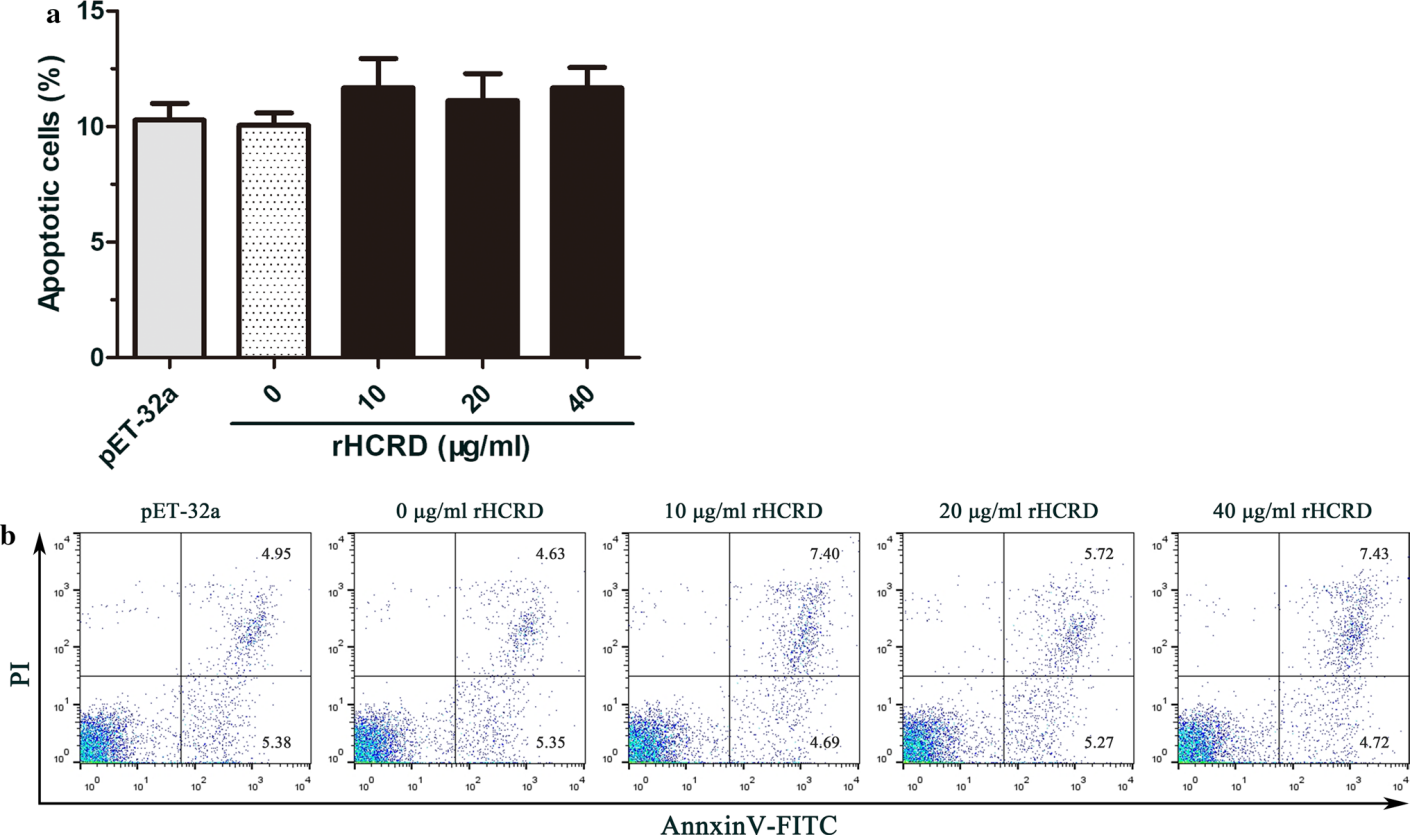
Recombinant rhodanese protein from *Haemonchus contortus* (rHCRD) did not induce apoptosis in goat peripheral blood mononuclear cells (PBMCs). PBMCs were cultured for 24 h with or without a range of concentrations of rHCRD and His-tagged protein. Propidium iodide (PI) and annexin V were used to stain the cells, which were subsequently analyzed by flow cytometry to quantify apoptotic cells. **a** Apoptotic cells (annexin V+/PI-) were plotted as a percentage of the total cell population. **b** Death of goat PBMCs after exposure to rHCRD is shown by a dot plot. Tests were independently performed in triplicate. (*P < 0.05, **P < 0.01, ***P < 0.001)

Apoptosis analysis showed that rHCRD had no significant effect on the apoptosis of goat PBMCs (10 μg/ml: *t*_(4)_ = 2.045, *P* = 0.1103; 20 μg/ml: *t*_(4)_ = 1.422, *P* = m0.2280; 40 μg/ml: *t*_(4)_ = 2.697, *P* = 0.0543). There was also no significant change in the His-tag protein-treated group (Fig. 5).

### Cytokine modulation

**Fig. 6:**
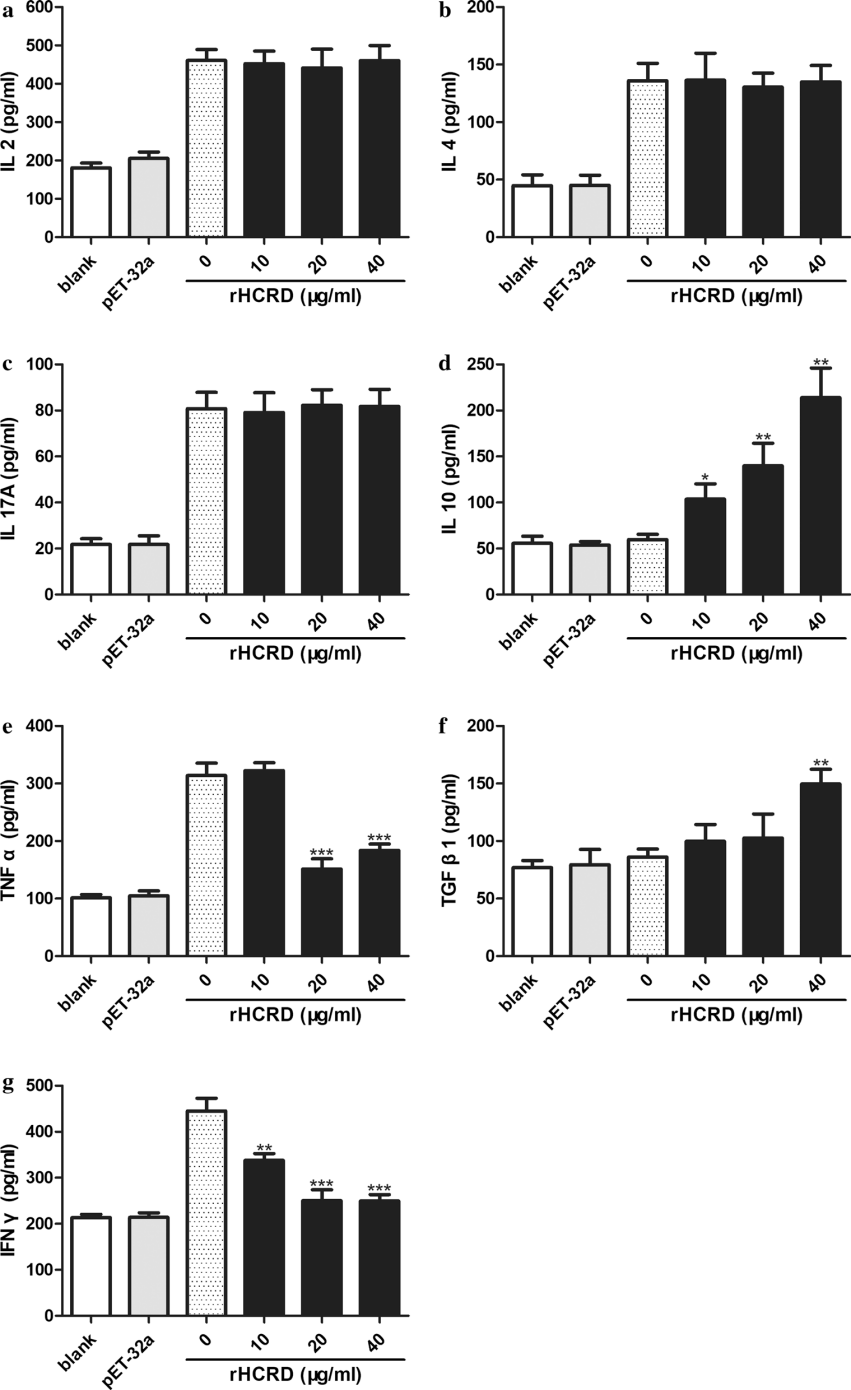
The cytokine profile of goat peripheral blood mononuclear cells (PBMCs) is modulated by recombinant rhodanese protein from *Haemonchus contortus* (rHCRD). Concanavalin A (ConA, 10 μg/ml) was used to stimulate goat PBMCs for 72 h with or without a series of concentrations of rHCRD and His-tagged protein. Enzyme-linked immunosorbent assays (ELISAs) were used to quantify cytokine secretion in the cell culture supernatant. **a** IL-2. **b** IL-4. **c** IL-17A. **d** IL-10. **e** TNF-α. **f** TGF-β1. **g** IFN-γ. Tests were independently performed in triplicate. (*P < 0.05, ** *P*< 0.01, ***P < 0.001)

ELISA results showed that, compared with ConA alone, rHCRD inhibited the expression of TNF-α (10 μg/ml: *t*_(4)_ = 0.5788, *P* = 0.5938; 20 μg/ml: *t*_(4)_ = 10.04, *P* = 0.0006; 40 μg/ml: *t*_(4)_ = 9.302, *P* = 0.0007) and IFN-γ (10 μg/ml: *t*_(4)_ = 5.920, *P* = 0.0041; 20 μg/ml: *t*_(4)_ = 9.255, *P* = 0.0008; 40 μg/ml: *t*_(4)_ = 10.88, *P* = 0.0004) and remarkably enhanced the secretion of IL-10 (10 μg/ml: *t*_(4)_ = 4.268, *P* = 0.0130; 20 μg/ml: *t*_(4)_ = 5.488, *P* = 0.0054; 40 μg/ml: *t*_(4)_ = 8.155, *P* = 0.0012) and TGF-β1 (10 μg/ml: *t*_(4)_ = 1.469, *P* = 0.2158; 20 μg/ml: *t*_(4)_ = 1.287, *P* = 0.2675; 40 μg/ml: *t*_(4)_ = 7.577, *P* = 0.0016) in goat PBMCs induced with ConA. However, rHCRD had no significant effect on IL-2, IL-4 or IL-17A production (Fig. 6).

### Phagocytosis ability of goat monocytes

**Fig. 7:**
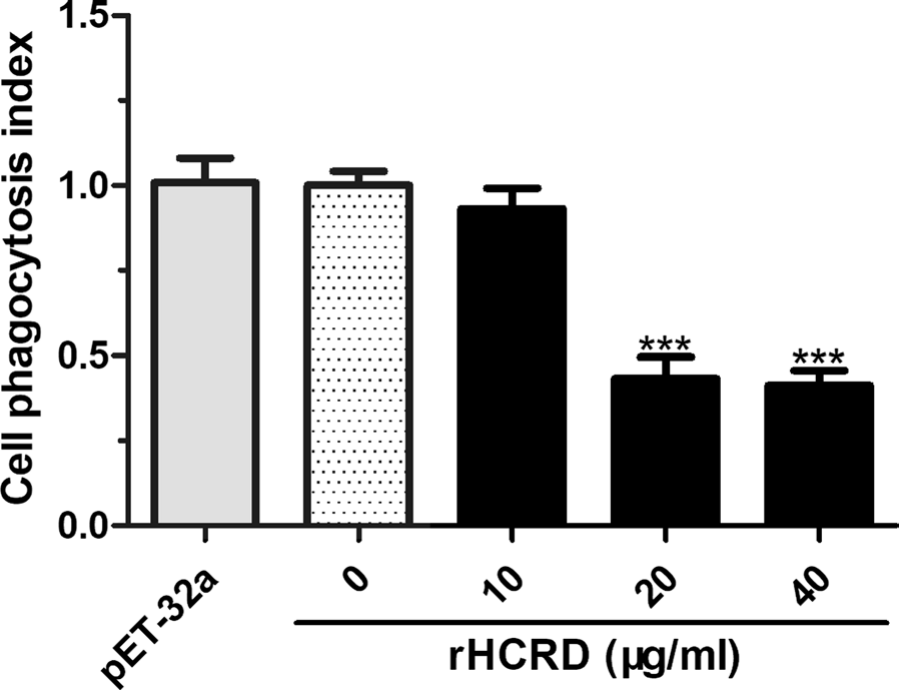
Inhibitory effect of recombinant rhodanese protein from *Haemonchus contortus* (rHCRD) on phagocytosis of goat monocytes. Cells were collected after rHCRD or his-tagged protein treatment for 48 h and incubated with FITC-dextran (1 mg/ml) for 1 h at 37 °C. The phagocytic activity of cells was analyzed on flow cytometry and calculated as mean fluorescence intensity (MFI). The data presented are results of three independent experiments (*P < 0.05, **P < 0.01, ***P < 0.001)

Goat monocytes were treated with different concentrations of rHCRD for 48 h, as shown in Fig. 7. The results of flow cytometry analysis showed that protein concentrations of 20 μg/ml and 40 μg/ml significantly decreased the FITC-dextran uptake ability of goat monocytes (10 μg/ml: *t*_(4)_ = 1.625, *P* = 0.1794; 20 μg/ml: *t*_(4)_ = 13.04, *P* = 0.0002; 40 μg/ml: *t*_(4)_ = 16.86, *P* < 0.0001), whereas no significant change was observed in the His-tagged protein treated group.

### MHC expression on goat monocytes

**Fig. 8:**
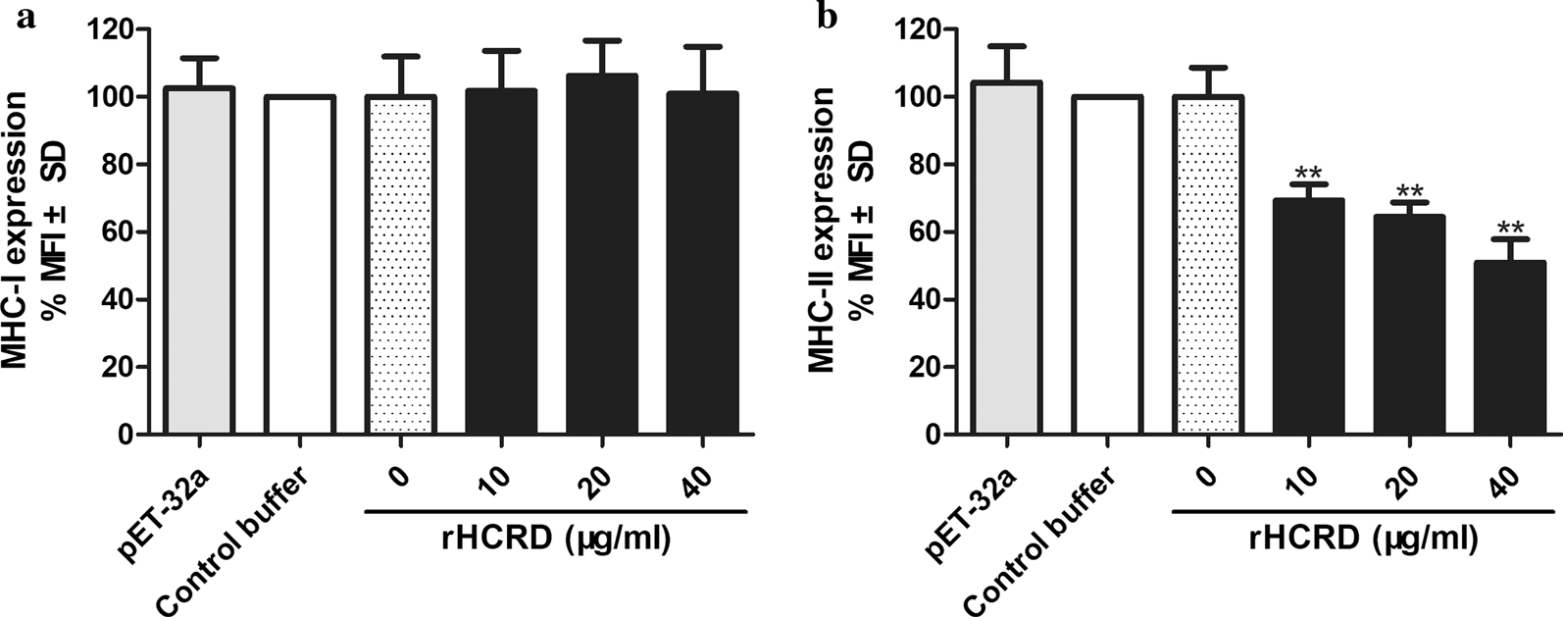
Inhibitory effect of recombinant rhodanese protein from *Haemonchus contortus* (rHCRD) on the expression of MHC-II by goat monocytes. Cells were cultured in the presence of varies rHCRD concentrations and his-tagged protein or control buffer (PBS/DTT) for 24 h. The cells treated with LPS were used as positive control. MHC-II expression was analysed on flow cytometric analysis and calculated as the percentage of mean fluorescence intensity (MFI) of controls. Bars represent the MFI ± SD of controls. The data presented are results of three independent experiments (*P < 0.05, **P < 0.01, ***P < 0.001). **a** MHC-I. **b** MHC-II

The results illustrated in Fig. 8, showed that rHCRD significantly decreased MHC-II expression in a dose-dependent manner as compared to the baseline expression of MHC-II in control buffer (10 μg/ml: *t*_(4)_ = 5.373, *P* = 0.0058; 20 μg/ml: *t*_(4)_ = 6.367, *P* = 0.0031; 40 μg/ml: *t*_(4)_ = 7.691, *P* = 0.0015), whereas, no significant change was observed in the His-tagged protein treated group. However, goat monocytes exposed to different concentrations of rHCRD did not show any change in MHC-I expression (10 μg/ml: *t*_(4)_ = 0.1773, *P* = 0.8679; 20 μg/ml: *t*_(4)_ = 0.6904, *P* = 0.5279; 40 μg/ml: *t*_(4)_ = 0.09299, *P* = 0.9304) (Fig. 8).

## Discussion

Excretory and secretory products diffuse or leak from the body of parasites or are actively exported through secretory pathways [40]. Modulation of the immune response of host by helminths involves the excretory and secretory products released by these parasites [41–43]. In the present study a rhodanese homologue was described from the parasitic nematode *H. contortus* for the first time. We found that rHCRD could be recognized by the antisera of goats experimentally infected with *H. contortus*, and the native HCRD protein was predominantly localized to the body surface and internal surface of the gut of the parasite. Furthermore, immunofluorescence assays revealed that rHCRD could bind to the surface of goat PBMCs *in vitro*.

Naive T cell activation by antigen-presenting cells (APCs) triggers adaptive immune responses and promotes the secretion of corresponding cytokines, resulting in both T-cell differentiation and the proliferation of additional T cells [44]. The phenomenon of diminished proliferation of peripheral T cells in response to filarial-specific antigens is called lymphocyte hypoproliferation and was previously demonstrated in filarial-infected humans [45]. Diliani et al. [46] demonstrated that draining lymph node cells and splenocytes from mice injected with *Ancylostoma ceylanicum* excretory/secretory products resulted in decreased proliferation in response to both species-specific antigens and mitogens. In the present study, rHCRD significantly suppressed ConA-stimulated goat PBMC proliferation in a dose-dependent manner.

Two *Onchocerca volvulus* excretory/secretory proteins, OvALT-2 and OvNLT-1, suppress antigen-specific T cell proliferation *via* the induction of cell apoptosis [47]. Extensive studies by many investigators have revealed that parasites/parasite antigens induce apoptosis in a number of different host immune cells, including T lymphocytes [48–52], B lymphocytes [53, 54], natural killer cells [55], dendritic cells [56, 57] and monocytes/macrophages [58–62]. In the present study, rHCRD had no significant effect on the apoptosis of goat PBMCs.

IL-2, TNF-α and IFN-γ are Th1 cytokines involved in cell-mediated immune responses, such as the inflammatory response [63]. IL-4 plays an important role in the activation, differentiation and proliferation of B lymphocytes and participates in antibody class switching to IgG and IgE [64]. Currently, IL-17A is considered to play a key role in driving inflammation and protective immunity at both mucosal and non-mucosal sites [65–67]. It was reported that intestinal nematode infection levels correlate with the production of both IL-10 and TGF-β by the host [68]. In a model of *Trichuris muris*-infected mice, IL-10 was shown to play a key role in controlling the inflammation caused by parasite infection and in the establishment of long-term infection [69–71]. T cell TGF-β signaling plays an essential role in the modulation of the mouse intestinal immune response to *Heligmosomoides polygyrus* infection by limiting mucosal Th1 and Th2 cytokine production and increasing IL-10 production [72]. Furthermore, the inhibition of filarial-specific T-cell proliferation can be reversed *in vitro* by antibodies against IL-10 and/or TGF-β [73, 74]. In the present study, incubation with rHCRD significantly increased the production of IL-10 and TNF-β1by ConA-stimulated goat PBMCs. However, rHCRD significantly decreased the secretion of TNF-α and IFN-γ, but had no significant effect on IL-2, IL-4 or IL-17A production. Therefore, the cytokines modulated by rHCRD are responsible for the induction of an anti-inflammatory response, which may be favorable for parasite survival.

Peripheral blood monocytes, which represent one of the major classes of APCs, play a crucial role in the innate response of vertebrate hosts to viral, fungal, bacterial and parasitic infections [55, 75]. Phagocytosis is the process by which unwanted cells or invading pathogens are efficiently removed from tissues and organs by professional phagocytes, predominantly macrophages [76]. In the present study, the phagocytic capacity of goat monocytes decreased significantly after treatment with different concentrations of rHCRD. MHC-II molecules are constantly expressed on the surface of APCs, permitting them to present extracellular antigens and initiate the adaptive immune response [77], and activation of APCs increases MHC-II expression [78]. In the present study, we observed that rHCRD was able to inhibit MHC-II expression on goat monocytes in a dose dependent manner.

## Conclusions

In conclusion, our results showed that rHCRD could be bound by goat PBMCs and exert its immunomodulatory effects on multiple aspects that might facilitate immune evasion by *H. contortus*. Our findings demonstrate that IL-10 and TGF-β1 were increased by rHCRD. However, PBMC proliferation, TNF-α and IFN-γ were decreased by rHCRD. Moreover, MHC-II expression and phagocytosis of monocytes were decreased by rHCRD. However, as these data are the result of *in vitro* experiments with recombinant rhodanese, the mechanism by which rhodanese acts during *H. contortus* infection *in vivo* requires further study.

## Data Availability

The datasets supporting the conclusions of this article are included within the article.

## Abbreviations

HCRD: *Haemonchus contortus* rhodanese
rHCRD: recombinant proteins of *Haemonchus contortus* rhodanese
PBMC: peripheral blood mononuclear cell
FITC: fluorescein-5-isothiocyanate
RHD: rhodanese homology domain
L3: third-stage larvae
ORF: open reading frame
RT-PCR: reverse transcription-polymerase chain reaction
IPTG: isopropyl-β-D-thiogalactopyranoside
SDS-PAGE: sodium dodecyl sulfate polyacrylamide gel electrophoresis
LAL: *Limulus* amoebocyte lysate
EU: endotoxin unit
ELISA: enzyme-linked immunosorbent assay
TBS: Tris-buffered saline
TBST: Tris-buffered saline containing 0.1% Tween-20
PBS: phosphate-buffered saline
HRP: horseradish peroxidase
DAB: diaminobenzidine
LPS: lipophosphoglycan
MFI: median fluorescence intensity
DAPI: 2-(4-amidinophenyl)-6-indole carbamidinedihydrochloride
ConA: concanavalin A
DMEM: Dulbecco’s modified Eagleʼs medium
PI: propidium iodide
IL-2: interleukin-2
IL-4: interleukin-4
IL-10: interleukin-10
IL-17A: interleukin-17A
TNF-α: tumor necrosis factor-α
IFN-γ: interferon-γ
TGF-β1: transforming growth factor-β1
